# Tongue reconstruction post partial glossectomy during the COVID-19 pandemic. A case report

**DOI:** 10.1016/j.amsu.2020.08.044

**Published:** 2020-09-06

**Authors:** Albaraa Y. Alsini, Suhail Sayed, Hadad Hussein Alkaf, Sherif K. Abdelmonim, Mohammed Ali Alessa

**Affiliations:** a Otolaryngology Resident, Department of Otolaryngology, Alhada Armed Forces Hospital, Taif, 26792, Saudi Arabia; b Currently rotating at King Abdullah Medical City, Makkah, 24246, Saudi Arabia; c Associate Consultant, Head and Neck Surgery, King Abdullah Medical City, Makkah, 24246, Saudi Arabia; d Associate Consultant, King Abdullah Medical City, Makkah, 24246, Saudi Arabia; e Department of Otolaryngology-Head & Neck Surgery, Faculty of Medicine, Ain Shams University, Cairo, Egypt; f Consultant Head & Neck Surgical Oncology, King Abdullah Medical City, Makkah, 24246, Saudi Arabia; g Otolaryngology Head & Neck Consultant, King Abdullah Medical City, Makkah, 24246, Saudi Arabia

**Keywords:** Case report, Partial glossectomy, Artificial graft, Acellular dermal matrix, Tongue reconstruction, COVID-19 prevention

## Abstract

**Introduction:**

The COVID-19 pandemic has necessitated temporary modifications in the current head and neck oncology treatment paradigm. Till date, no definite treatment for COVID-19 has been discovered. Considering the situation of the global COVID-19 outbreak, methods that minimize patient visits with no compromise in efficacy should be considered. The optimal method for tongue reconstruction has not been determined yet. The artificial bilayer membrane has been used as mucosal substitute in few cases of tongue reconstruction with promising results.

**Case presentation:**

We present two cases of tongue reconstruction with acellular dermal matrix post partial glossectomy for tongue carcinoma during the COVID-19 pandemic. Both patients showed good recovery and healing, and no side effects and/or complications were reported.

**Discussion:**

The acellular dermal matrix is not a standard technique for tongue reconstruction but one of the available options. The few reported cases in literature showed promising results in regard of function and healing.

**Conclusion:**

We believe the use of acellular dermal matrix can help in preventing the spread of COVID-19 because of the absence of donor morbidity, decreasing post-operative hospital stay and visits.

## Highlights

• ADM is a useful alternative for tongue reconstruction instead of skin graft.
• ADM use in reconstruction will avoid skin graft doner site morbidity.
• ADM is safe to be used in oral cavity after partial glossectomy.
• ADM maybe better than skin graft further study need to be follow.

## 1 Introduction

Squamous cell carcinoma of the tongue can cause significant tissue and/or bone invasion and that may require reconstructive surgery after surgical excision. Reconstruction can improve patient quality of life and organ function considerably [1]. Reconstruction options for mucosal defects included primary closure, mucosal and split-thickness skin grafts, pedicled flaps, and microvascular free tissue transfer. [[2], [3], [4]] Recently, the uses of acellular dermal matrix (ADM) in variable head and neck reconstructions have been documented. [[5], [6], [7], [8]] ADM has several advantages such as promoting rapid neovascularization and normal mucosalization, absence of donor site morbidity, reduced surgical time, decreased duration of hospital stay, and minimum hospital visits [9]. Considering the situation of the global COVID-19 outbreak, methods that minimize patient visits with no compromise in efficacy should be considered. Hence, the advantages of ADM are particularly important in the current scenario to minimize the spread of COVID-19 as by decreased the frequency of contact between the health care providers and general population or patients.

Herein, according to SCARE guidelines [10]. we report the cases of two patients with partial glossectomy reconstructed with ADM during the COVID-19 pandemic, which might help in visualizing ADM as an alternative to other reconstructive options especially during the COVID-19 pandemic.

### 1.1 Case report 1

**Fig. 1:**
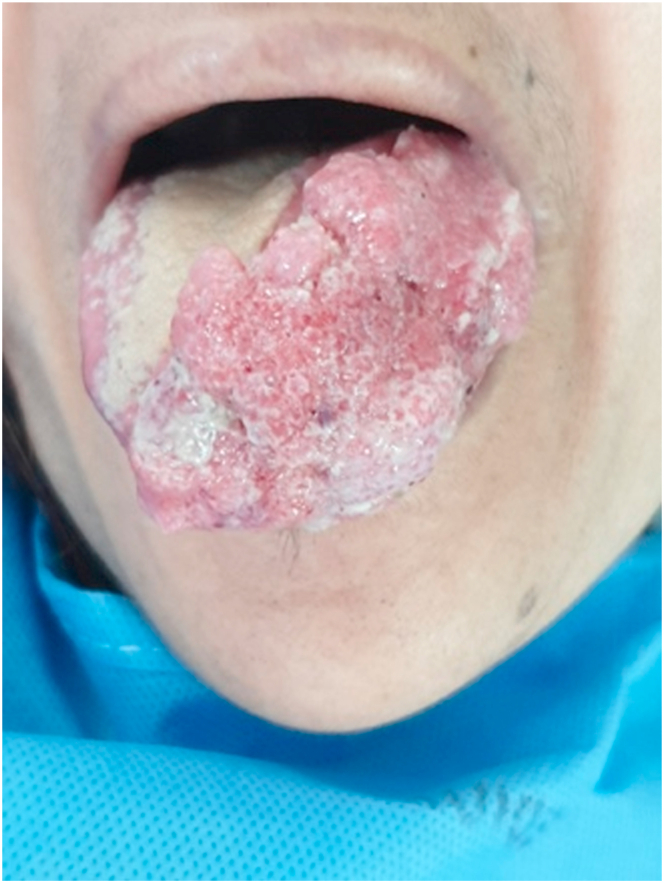
Left lateral fungating mass, extending to the tip.

**Fig. 2:**
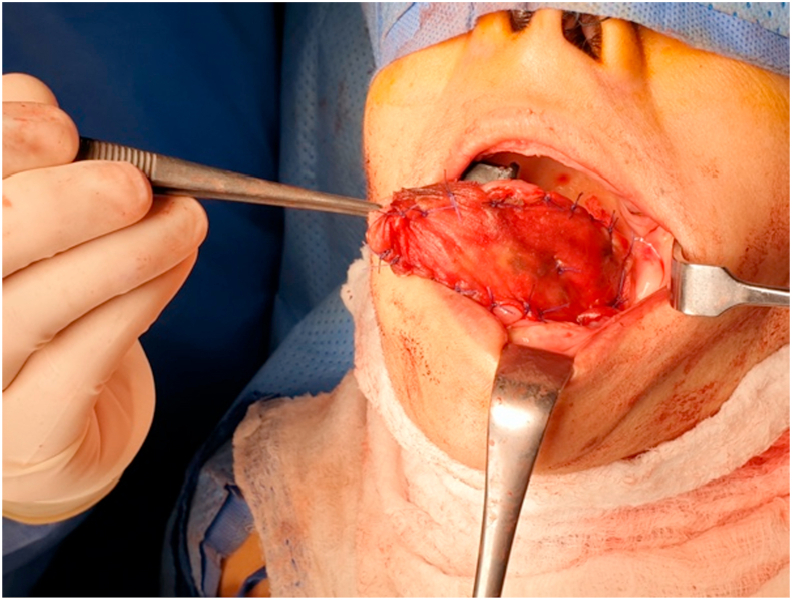
ADM designed and used to reconstruct the surgical defect.

**Fig. 3:**
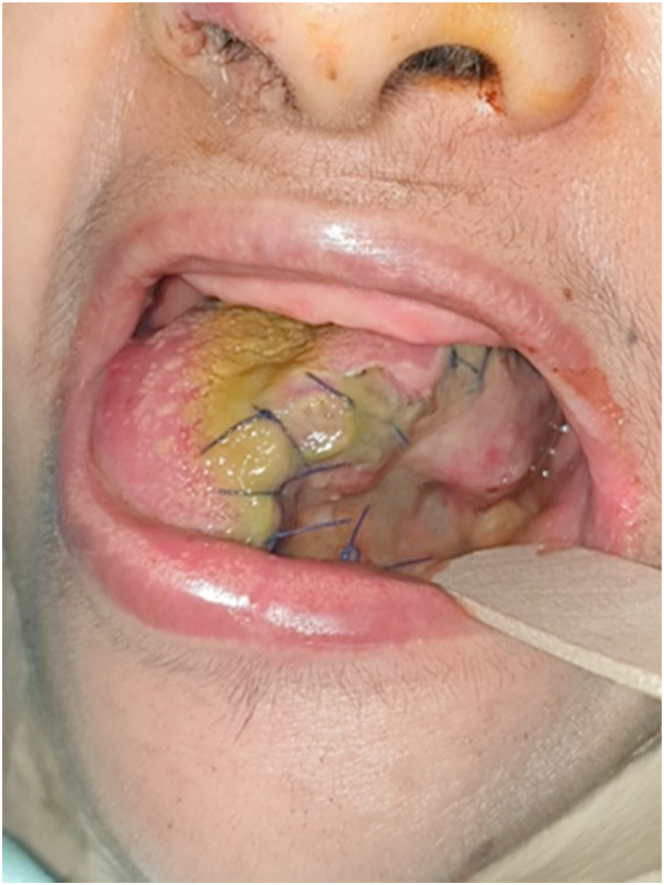
Day 2; Showed healing started with no bleeding or infection.

The patient was a 54-years-old white women with known history of hypertension and diabetes mellitus on oral hypoglycemic medications. She was referred from a peripheral hospital to our tertiary center with the diagnosis of squamous cell carcinoma (SCC) of the tongue. The patient complained of feeling a mass in the tongue for 6 months, which increased in size over time, bled on touch, and was associated with difficulties in speech and mastication. She did not report any previous history of cancer, exposure to radiations, smoking and/or alcohol consumption. The patient denied family history of oral cavity carcinoma. On intraoral examination, a hard fungating mass was observed over the left lateral part of the tongue extending to the tip “(shown in Fig. 1)”. Additionally, poor oral hygiene and the presence of a poor denture was observed. Other parts of the oral cavity were grossly normal, including the floor of mouth. On neck examination, palpable level II lymph nodes were evident on the left side; otherwise, no significant findings were observed. Findings from examinations of the ear, nose, and cranial nerves were unremarkable. Flexible pharyngolaryngoscopy did not reveal any pathological mass or suspicious area; vocal cords were mobile bilaterally. The biopsy slides from the referred hospital were reviewed in our institution and the observed tissue was diagnosed as well differentiated SCC. Standard cancer and metastasis examinations were performed, and the case was discussed in the multidisciplinary clinic. The cancer was staged as cT3N0M0 as per the criteria of the 8th edition of the American Joint Committee on Cancer (AJCC). The patient was started on prophylactic cefazolin before the surgery. Bilateral level I-IV neck dissection and partial glossectomy were performed using monopolar electrocautery including all margins determined negative on frozen sections. The tongue was reconstructed using ADM, which is designed to fit the defect, placed in the defect, and fixed by simple sutures to the defect edges and two simple stitches at the base of the ADM to minimize the graft movement till healing occur. We used 4.0 Vicryl absorbable sutures “(shown in Fig. 2)”. The tongue reconstruction took 10 minutes duration. The procedure performed by head and neck surgeon consultant. The patient showed uneventful post-operative recovery. Post-operative care included administration of analgesics and prophylactic antibiotics, antiseptic gargles, and nil per-oral for first 24 hours. On the next day, clear liquid diet was initiated, and the patient was advised to avoid citrus juices or milk products. Two days after the surgery “(shown in Fig. 3)”, initiation of soft diet was advised. Subjective evaluation of pronunciation and sounds was reported to be better, compared to the pre-operative condition. The patient was discharged at the 7th day post-operative day, and wound healing was satisfactory with good oral intake.

**Fig. 4:**
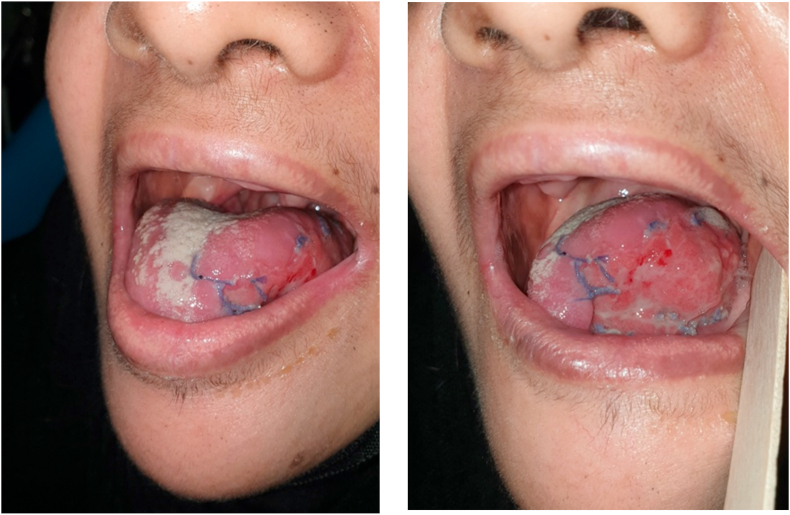
At one month follow up, normal mucosalization & good bulk.

The final histopathology report staged the tumor as pT3N0M0 as per 8th edition of the AJCC; both tumor size and depth of invasion (DOI) were 6 cm, 6 mm respectively. Patient received post-operative radiotherapy. Till date of this report, patient satisfaction was good at the 5-month follow-up” (Fig. 4 a,b at 1 month follow up)” in terms of mastication, speech, and healing. Tongue movement has been assessed at 5th month follow up and no limitation noticed. We assessed the efficacy of this technique subjectively by significant improving the pronunciation, tongue mobility without restriction and tongue bulk. Unfortunately, we did not use objective tools for such assessment.

### 1.2 Case report 2

The patient was a 75-years-old white women with known history of diabetes mellitus, hypertension, and asthma. She reported a previous history of carcinoma of the left side of the tongue, which was excised completely 5 years ago. She was not given any adjuvant therapy at the time. Currently, the patient complained of feeling a mass in the tongue since 3 months, which increased in size with time and was associated with pain and bleeding. No history of exposure to radiation was reported. She was not an alcoholic or a smoker. The patient denied family history of oral cavity carcinoma. On intraoral examination, a fungating lesion (approximately 2 × 3 cm) was observed at the left lateral part of the tongue, which was hard and did not involve the floor of mouth. Other subsites of the oral cavity did not show any remarkable findings. The patient showed poor oral hygiene.

Neck examination did not the presence of any palpable masses. Findings from complete head and neck examination with a flexible scope were unremarkable. Biopsy of the lesion was performed, which revealed invasive, moderately differentiated SCC.

**Fig. 5:**
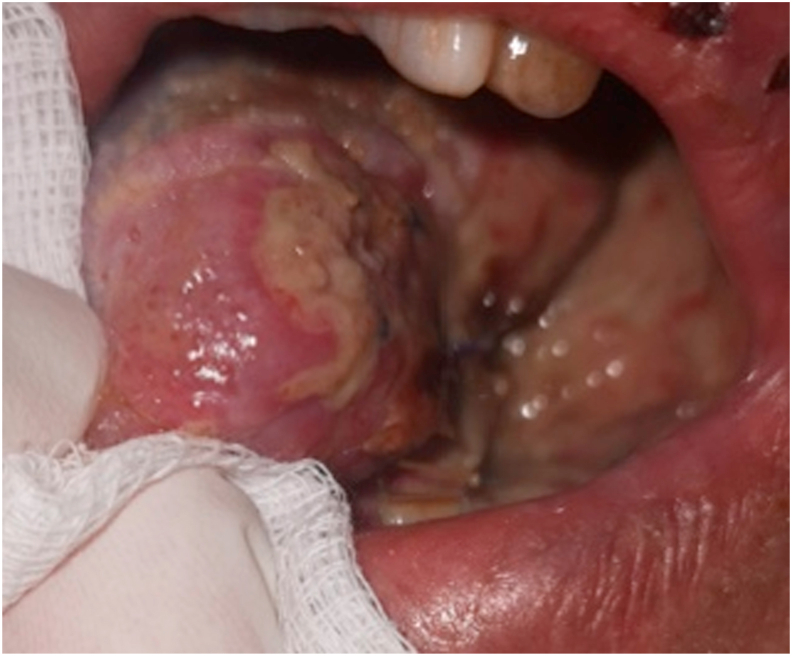
Day 7th post tongue reconstruction, showed healing started, no bleeding or infection.

Standard cancer and metastatic examinations were performed, and the case was discussed in the multidisciplinary clinic. The carcinoma was staged as cT2N0M0 as per the 8th edition of the AJCC. Routine course of antibiotic prophylaxis with cefazolin was initiated. Type III modified radical neck dissection and supraomohyoid neck dissection on left and right side, respectively, with partial glossectomy using monopolar electrocautery were performed including safe margins determined by frozen sections. The tongue defect was reconstructed using ADM, which was cut to fit the defect and was fixed by simple sutures to the defect edges and tow simple stitches at the base of the ADM to minimize the graft movement till healing occur. We used 4.0 Vicryl absorbable sutures. The tongue reconstruction took 10 minutes duration. The procedure performed by head and neck surgeon consultant. The patient showed uneventful post-operative recovery. Post-operative care was same as that for case 1. The final pathology report staged the carcinoma as pT3N2bM0 as per the 8th edition of the AJCC. Pathological T3 was staged in this case, as the DOI was 12 mm, and tumor size was 2.5 cm. The patient was discharged in good condition and wound healing at the 7th post-operative day” (shown in Fig. 5)”. Patient received post-operative radiotherapy. Till date of this report, patient satisfaction was good at the 4th month follow-up in terms of mastication, speech, and healing. Tongue movement has been assessed at 5th month follow up and no limitation noticed. We assessed the efficacy of this technique subjectively by significant improving the pronunciation, tongue mobility without restriction and tongue bulk. Unfortunately, we did not use objective tools for such assessment.

## 2 Discussion

During COVID-19 pandemic, it is crucial to protect healthcare providers from COVID-19 to ensure continuity of the health care system [11]. We believe uses of ADM for tongue reconstruction, as in our reports, may add a value in COVID-19 prevention as by minimizing the number and duration of non-essential hospital visits & stay post-operatively. The ADM product we used is composed of bovine type I collagen and elastin, it promotes rapid cell migration, proliferation and revascularization. The ADM we used is from Germany, and for unknown reason the product is not available in United States market as it provided in the company website.

Generally, surgery results in superior outcomes in contrast to other treatment modalities in early stage oral carcinoma. However, methods for optimal reconstruction of the oral defects are still controversial. A small defect can be reconstructed with split thickness skin graft (STSG) while larger defects may require microvascular free tissue transfer flaps [12]. Glossectomy is often associated with tissue loss and distortion of speech and mobility of the tongue. The efficacy of synthetic collagen material in tongue reconstruction after partial glossectomy has been studied in few cases. The ADM is an option for reconstruction of tongue defects, and it can prevent the sequalae of fibrosis and scarring associated with STSG, preserving organ function. It is not a standard technique of tongue reconstruction but one of the available options. The potential advantages of ADM include reduced surgical time, absence of donor site morbidity, rapid neovascularization, and hence, reduced hospital stays [9]. Such decreases in non-essential hospital visits and stay can potentially decrease the spread of COVID-19. Hence, it should be considered as one of the alternatives for tongue reconstruction in this pandemic period, and it continued use should also be supported in future. Rhee et al., used ADM to reconstruct oral defects at different sites including the tongue in 29 patients and reported a success rates of 90% in an average follow-up duration of 8.6 months. Tahim et al. reported five cases reconstructed by collagen scaffold material post partial glossectomy, and the results after 6 weeks were satisfactory in terms of restoration of function and healing [13].

In one case, biological dural graft was used to reconstruct the tongue, and at one-year follow-up, the patient had regained normal articulation, and healing was adequate [14].

The overall speech intelligibility score of patients reconstructed using artificial bilayer membrane as a mucosal substitutes after partial glossectomy ranged from 86% to 97% as reported by Terai et al.,[14] Girod et al. evaluated the efficacy of reconstructions with STSG and ADM in restoration of function. The authors concluded that ADM results in better functional outcomes and significant cost reduction in compared to STSG, in addition to the advantages mentioned by previous authors. In term of histopathology, reconstructions using ADM showed lesser amount of fibrosis and inflammation, though the collagen content is similar. The long-term side effects of radiotherapy were similar for reconstructions with STSG and ADM, although functional restoration post radiotherapy was better with ADM compared to that with STSG [9]. In our cases, we used ADM for tongue reconstruction after partial glossectomy and consider it a useful alternative especially during the COVID-19 pandemic. However, long-term follow-ups were not performed for both patients, although healing at one-month follow-up in case 1 was good with no limitations in tongue mobility. The candidates have full right sharing their experience at any time during and/or after treatment. In addition to the promising results of previous studies demonstrating it to be a safe and efficient treatment option, we suggest that it should be especially considered for reconstructions during the COVID-19 global outbreak.

## Funding source

No financial or funding support.

## Ethical Approval

Written informed consent was obtained from both patients for publication of this case report and accompanying images.

## Consent

Written informed consent was obtained from both patients for publication of this case report and accompanying images. A copy of the written consent is available for review by the Editor-in-Chief of this journal on request.

## Author contribution

All authors; Albaraa Y. Alsini, Suhail Sayed, Hadad Hussein Alkaf, Sherif K. Abdelmonim, Mohammed Ali Alessa, had involved in study design, data collection, reviewed the literature, manuscript preparation, and we reviewed the final version of the project and all agreed for publication.

## Registration of Research Studies

Name of the registry:

Unique Identifying number or registration ID:

Hyperlink to your specific registration (must be publicly accessible and will be checked):

## Guarantor

Mohammed Ali Alessa, Otolaryngology Head & Neck Consultant, King Abdullah Medical City, Makkah, Saudi Arabia.

## Declaration of competing interest

Authors have no conflicts of interest.
